# The outcomes and prognostic factors of acute respiratory failure in the patients 90 years old and over

**DOI:** 10.18632/oncotarget.24051

**Published:** 2018-01-09

**Authors:** Wan-Ling Chen, Chin-Ming Chen, Shu-Chen Kung, Ching-Min Wang, Chih-Cheng Lai, Chien-Ming Chao

**Affiliations:** ^1^ Department of Respiratory Therapy, Chi Mei Medical Center, Tainan, Taiwan; ^2^ Department of Intensive Care Medicine, Chi Mei Medical Center, Tainan, Taiwan; ^3^ Department of Recreation and Health-Care Management, Chia Nan University of Pharmacy and Science, Tainan, Taiwan; ^4^ Department of Internal Medicine, Chi Mei Medical Center, Tainan, Taiwan; ^5^ Department of Intensive Care Medicine, Chi Mei Medical Center, Tainan, Taiwan; ^6^ Department of Nursing, Min-Hwei College of Health Care Management, Tainan, Taiwan

**Keywords:** aged, 90 and over, mechanical ventilation, outcome, risk factor

## Abstract

This retrospective cohort study investigated the outcomes and prognostic factors in nonagenarians (patients 90 years old or older) with acute respiratory failure. Between 2006 and 2016, all nonagenarians with acute respiratory failure requiring invasive mechanical ventilation (MV) were enrolled. Outcomes including in-hospital mortality and ventilator dependency were measured. A total of 173 nonagenarians with acute respiratory failure were admitted to the intensive care unit (ICU). A total of 56 patients died during the hospital stay and the rate of in-hospital mortality was 32.4%. Patients with higher APACHE (Acute Physiology and Chronic Health Evaluation) II scores (adjusted odds ratio [OR], 5.91; 95 % CI, 1.55-22.45; p = 0.009, APACHE II scores ≥ 25 vs APACHE II scores < 15), use of vasoactive agent (adjust OR, 2.67; 95% CI, 1.12-6.37; p = 0.03) and more organ dysfunction (adjusted OR, 11.13; 95% CI, 3.38-36.36, p < 0.001; ≥ 3 organ dysfunction vs ≤ 1 organ dysfunction) were more likely to die. Among the 117 survivors, 25 (21.4%) patients became dependent on MV. Female gender (adjusted OR, 3.53; 95% CI, 1.16-10.76, p = 0.027) and poor consciousness level (adjusted OR, 4.98; 95% CI, 1.41-17.58, p = 0.013) were associated with MV dependency. In conclusion, the mortality rate of nonagenarians with acute respiratory failure was high, especially for those with higher APACHE II scores or more organ dysfunction.

## INTRODUCTION

The aging population is rapidly increasing. The World Health Organization (WHO) estimates that by 2050, 1.5 billion people, representing 16% of the global population, will be 65 years old and older. With the increases in the aging population and patients with multiple comorbidities, more patients will require invasive mechanical ventilator (MV) for management of acute respiratory failure [1–3]. Even if elderly patients survive acute illness, they are at high risk of prolonged MV [4–7]. However, these very elderly patients are near the end of life and many may prefer preserving quality of life rather than prolonging survival [8–10]. Thus, this specific population may be reluctant to accept unnecessary prolongation of life by life-sustaining therapy such as invasive MV [10, 11]. Therefore, a study to investigate the outcomes and prognostic factors in very elderly patients with acute respiratory failure requiring MV is warranted. After the outcomes can be fully understood and with an accurate prognosis, physicians can help patients and their families make the best choice regarding MV. Currently, there is limited information on the outcomes and prognosis in very elderly patients who require invasive MV [12, 13]. The aims of this study were to investigate the outcomes of nonagenarians (patients 90 years old or older) with acute respiratory failure requiring invasive MV and to identify risk factors associated with mortality.

## RESULTS

### Patient characteristics

During the eleven-year period, a total of 173 nonagenarians with acute respiratory failure using MV were admitted to the ICU. Among them, the mean age of the patients was 92.1 ± 2.2 years (range: 90 −99 years). Women comprised most of the patients (n =92, 53.2%). Most patients were admitted to the medical ICU (n = 126, 72.8%), and the others were admitted to the surgical ICU (47, 27.2%). Sepsis was the most common diagnosis of acute respiratory failure (n = 66, 38.2%), followed by gastrointestinal and hepatobiliary disease (n = 35, 20.2%), respiratory disease (n = 28, 16.2%), cardiovascular disease (n = 19, 11.0%), neurologic disease (n = 13, 7.5%), endocrine disease (n = 2, 1.2%), renal disease (n = 1, 0.6%), and others (n = 9, 5.2%). Hypertension was the most common underlying disease (n = 115, 66.5%), followed by chronic kidney disease (n = 45, 26.0%), stroke (n = 41, 23.7%), diabetes mellitus (n = 39, 22.5%), congestive heart failure (n = 36, 20.8%), chronic obstructive pulmonary disease (n = 35, 20.2%), dementia (n = 26, 15.0%) and malignancy (n = 24, 13.9%). In addition, 33 patients (19.1%) had a recent history of corticosteroid administration. Respiratory dysfunction was the most common organ dysfunction (n = 148, 85.5%), followed by cardiovascular (n = 81, 46.8%), renal (n = 63, 36.4%), liver (n = 21, 12.1%) and hematologic organ dysfunction (n = 12, 6.9%). Dysfunction of at least one organ was found in 155 (89.6%) patients. During ICU hospitalization, seven patients had continuous renal replacement therapy and six patients used intermittent hemodialysis. One patient had an intra-aortic balloon pump for hemodynamic support. Eleven patients received a tracheostomy. Ninety-two (53.2%) patients were successfully weaned from MV, but 25 (14.5%) patients became MV-dependent. The LOS in the ICU and hospital was 8.5 ± 6.8 (median: 8 days, IQR: 3-12) and 24.6±25.4 days (median, 18 days, IQR: 10-33), respectively.

### Outcome analysis

A total of 56 patients died during the hospital stay and the rate of in-hospital mortality was 32.4%. We further compared the clinical variables of patients with survived and those who died (Table 1). Patients who died had lower Glasgow coma scores, higher APACHE II scores, more underlying chronic kidney disease, poorer renal function, more frequent use of vasoactive agents and more cardiovascular, hematologic, renal and liver failure than the survivors (Table 1). In contrast, the survivor had more tracheostomy, and shorter length hospital stay (Table 1). Table 2 summarizes the risk factors associated with the in-hospital mortality rate using stepwise logistic regression analysis. Patients with higher APACHE II scores, use of vasoactive agent and more organ dysfunction were more likely to die (Table 2).

**Table 1:** The comparison of clinical variables between patients with survival and mortality outcomes

Variables	No (%) of patients with survival outcomes (*n*=117)	No (%) of patients with mortality outcomes (*n*=56)	P value
Age (years)	92.1 ± 2.3	92.0 ± 2.0	0.68
Gender			
Male	54 (46.2)	27 (48.2)	0.80
Female	63 (53.8)	29 (51.8)	
Category of ICU			0.66
Medical ICU	84 (71.8)	42 (75.0)	
Surgical ICU	33 (28.2)	14 (25.0)	
Glasgow Coma Scale	11.0 (9.0-14.0)	7.0 (3.0-11.0)	< 0.001
APACHE II scores	15.0 (12.0-20.0)	24.0 (18.0-34.0)	< 0.001
Comorbidity			
Dementia	21 (17.9)	5 (8.9)	0.12
Hypertension	82 (70.1)	33 (58.9)	0.15
COPD	23 (19.7)	12 (21.4)	0.79
CHF	21 (17.9)	15 (26.8)	0.18
Stroke	30 (25.6)	11 (19.6)	0.39
Diabetes mellitus	23 (19.7)	16 (28.6)	0.19
CKD	24 (20.5)	21 (37.5)	0.017
Liver cirrhosis	2 (1.7)	3 (5.4)	0.33
Malignancy	16 (13.7)	8 (14.3)	0.91
Receiving steroid	24 (20.5)	9 (16.1)	0.49
Laboratory examinations			
BUN (mg/dl)	26.0 (19.0-37.0)	30.5 (19.8-54.3)	0.07
Creatinine(mg/d)	1.2 (0.9-1.6)	1.6 (1.1-2.4)	0.009
Hemoglobin (g/dl)	11.2 (9.8-13.1)	10.9 (9.6-12.2)	0.50
Albumin (g/dl)	2.8 (2.4-3.2)	2.5 (2.2-2.9)	0.053
Requiring hemodialysis			
IHD	4 (3.4)	2 (3.6)	1.0
CRRT	2 (1.7)	5 (8.9)	0.37
Use of vasoactive agents	24 (20.5)	33 (58.9)	<0.001
Patients received tracheostomy	11 (9.4)	0 (0.0)	0.02
Organ dysfunction			
Respiratory system	96 (82.1)	52 (92.9)	0.059
Cardiovascular system	31 (26.5)	50 (89.3)	< 0.001
Hematologic system	1 (0.9)	11 (19.6)	< 0.001
Renal system	30 (25.6)	33 (58.9)	< 0.001
Liver system	10 (8.5)	11 (19.6)	0.037
Lengths of ICU stay	8.0 (5.0-12.0)	5.0 (1.3-12.0)	0.06
Lengths of hospital stay	22.0 (13.0-39.5)	8.0 (2.0-23.3)	<0.001

Data expressed as number (%), mean ± standard deviation (SD) or median with interquartile range (IQR).

Abbreviations: ICU, intensive care unit; APACHE II, Acute Physical and Chronic Healthy Evaluation II; COPD, chronic obstructive pulmonary disease; CHF, congestive heart failure; CKD, chronic kidney disease; IHD, intermittent hemodialysis; CRRT, continuous renal replacement therapy.

**Table 2:** Risk factor of in-hospital mortality

Variable	Crude OR	95% CI	P value	Adjust OR	95% CI	P value
**APACHE II score**						
< 15	1.00			1.00		
15-24	2.73	1.16-6.4	0.021	2.21	0.79-6.13	0.13
≥25	12.00	4.67-30.85	<0.001	5.91	1.55-22.45	0.009
**Glasgow Coma Scale**						
≥ 8	1.00			1.00		
< 8	4.39	2.19-8.79	<0.001	1.41	0.49-4.00	0.53
**Underlying chronic kidney disease**						
No	1.00			1.00		
Yes	2.32	1.15-4.70	0.019	1.42	0.57-3.58	0.45
**Vasoactive agent**						
No	1.0			1.0		
Yes	5.56	2.77-11.15	<0.001	2.67	1.12-6.37	0.03
**Number of organ dysfunction**						
≤ 1	1.00			1.00		
2	4.85	1.76-13.35	0.002	2.90	0.94-8.89	0.06
≥3	25.54	8.99-72.54	<0.001	11.13	3.38-36.63	<0.001

Abbreviations: APACHE II, Acute Physical and Chronic Healthy Evaluation II; OR, odds ratio.

Among the 117 survivors, 25 (21.4%) patients became MV-dependent. These patients were more like to be women, have lower Glasgow Coma Scale scores, have higher APACHE II scores, more use of vasoactive agents and more respiratory organ dysfunction than the patients with successful weaning (Table 3). They also had significantly longer LOS in the ICU and hospital than patients with successful weaning. After stepwise logistic regression analysis, we identified two independent risk factors – gender and consciousness level - which were associated with MV dependence. Women had a higher risk of becoming ventilator-dependent than men (adjusted OR, 3.53; 95% CI, 1.16-10.76, p = 0.027). Patients with Glasgow Coma Scale scores < 8 had a higher risk of becoming ventilator-dependent than those with higher scores (adjusted OR, 4.98; 95% CI, 1.41-17.58, p = 0.013).

**Table 3:** The comparison of clinical variables between patients with successful weaning and mechanical ventilation-dependent

Variables	No (%) of patients with successful weaning (*n*=92)	No (%) of patients with MV-dependent (*n*=25)	P value
Age (years)	92.0 ± 2.2	92.5 ± 2.5	0.40
Gender			0.012
Male	48 (52.2)	6 (24.0)	
Female	44 (47.8)	19 (76.0)	
Glasgow Coma Scale	12 (10 -14)	9 (6 -10)	< 0.001
APACHE II scores	19 (13 - 30)	16 (13 - 23)	0.013
Comorbidity			
Dementia	17 (18.5)	4 (16.0)	0.78
Hypertension	63 (68.5)	19 (76.0)	0.47
COPD	19 (20.7)	4 (16.0)	0.60
Congestive heart failure	18 (19.6)	3 (12.0)	0.38
Stroke	21 (22.8)	9 (36.0)	0.18
Diabetes mellitus	18 (19.6)	5 (20.0)	0.96
Chronic kidney disease	21 (22.8)	3 (12.0)	0.24
Liver cirrhosis	1 (1.1)	1 (4.0)	0.38
Malignancy	13 (14.1)	3 (12.0)	0.78
Receiving steroid	19 (20.7)	5 (20.0)	0.94
Use of vasoactive agents	15 (16.3)	9 (36.0)	0.03
Organ dysfunction			
Respiratory system	72 (78.3)	24 (96.0)	0.04
Cardiovascular system	23 (25.0)	8 (32.0)	0.48
Hematologic system	0 (0.0)	1 (4.0)	0.21
Renal system	24 (26.1)	6 (24.0)	0.83
Liver system	8 (8.7)	2 (8.0)	0.91
ICU length of stay (days)	8.0 (4.3 -11.0)	12.0 (8.0 -15.0)	0.002
Length of hospital stay (days)	19.0 (13.0 - 30.0)	45.0 (18.0 - 56.5)	0.001

Data expressed as number (%), mean ± standard deviation (SD) or median with interquartile range (IQR).

Abbreviations: APACHE II, Acute Physical and Chronic Healthy Evaluation II; COPD, chronic obstructive pulmonary disease; ICU, intensive care unit.

## DISCUSSION

To the best of our knowledge, this is the first study to investigate the clinical outcomes of nonagenarians with acute respiratory failure and there were several significant findings. First, the outcome of these very elderly patients with acute respiratory failure was poor and the in-hospital mortality rate was high at 32.4%. Similar findings have been noted in several studies with all-cause hospital mortality rates for critically ill patients 80 years old and older admitted to the ICU ranging from 26% to 50% [14–16]. About one-fifth of survivors become ventilator-dependent. In summary, only half of these patients can survive to discharge and be weaned successfully from MV. Because age itself cannot be the only criterion for health care decision-making, information regarding the prognosis of nonagenarians with acute respiratory failure is important for communication between intensivists and patients and their families [17]. If enough information on outcomes can be obtained, communication between physicians and patients and family can be enhanced and decisions about aggressive management or palliative care can be made appropriately for these elderly critical ill patients [17–19].

Second, previous studies [20–22] have shown that APACHE II scores and number of failed organs are independent predictors of hospital outcome among various populations with acute respiratory failure. In this study of very elderly patients, these two risk factors were found to be independently associated with mortality. Among twenty-six of the 39 (66.7%) patients with APACHE II scores≥ 25 died. and 14 of the 16 (87.5%) patients with ≥ 3 dysfunctional organs died. Our findings indicated that higher APACHE II scores and more organ dysfunction resulted in higher mortality. In addition, use of vasoactive agents was found to be associated with in-hospital mortality (adjusted OR, 2.67; 95% CI, 1.12-6.37, p = 0.03) and it suggests that the initial hemodynamic status should be a good indicator of poor outcome among these elderly patients with acute respiratory failure. End of life care should be considered when treating these patients with poor prognoses.

Third, this study also aimed to determine the risk factors for MV dependency in nonagenarians with acute respiratory failure. Poor consciousness level was independently associated with the risk of ventilator dependency. This is a reasonable association, as an unclear consciousness can result in difficult clearance of secretions and difficult airway maintenance. Moreover, the resolution to difficult airway maintenance – tracheostomy- was only performed in 11 (6.4%) patients in this study. All of these reasons can cause ventilator dependence. Additionally, female gender was associated with a higher risk of ventilator dependency than male gender in this study. The gender difference is difficult to explain and requires further investigation.

This study had several limitations. First, only two major outcomes, in-hospital mortality and ventilator-dependency, were measured. We did not assess long- term outcomes or the functional status of patients after discharge. Further study is warranted to investigate these issues. Second, our findings were based on a single institution. Therefore, it may not be generalized to other hospitals or countries. Finally, we did not collect the data regarding the use of sedative and analgesic agents in this study for analysis. However, these variables may be possible confounding factors.

In conclusion, the mortality rate of nonagenarians with acute respiratory failure was high, especially for patients with higher APACHE II scores or more organ dysfunction. In addition, a significant percentage of patients became ventilator-dependent, especially for patients with a poor consciousness level.

## MATERIALS AND METHODS

### Patients and hospital setting

This study was conducted in the retrospective cohort observation design. Chi Mei Medical Center is a 900-bed regional hospital with 63 intensive care unit (ICU) beds. Between January, 2006 and December, 2016, all patients 90 years old or older with acute respiratory requiring invasive MV were identified. The medical records of all nonagenarians with invasive MV were retrospectively reviewed and the following information was collected: age, gender, the category of ICU, length of stay (LOS) in the ICU and hospital, Acute Physical and Chronic Healthy Evaluation II (APACHE II) score, Glasgow Coma Scale score, serum albumin, hemoglobin, blood urea nitrogen (BUN), and creatinine levels and comorbidities, including dementia, hypertension, chronic obstructive pulmonary disease, congestive heart failure, chronic kidney disease, diabetes mellitus, liver cirrhosis, stroke, and cancer. The main reason for mechanical ventilation was categorized as follows: respiratory disease (including asthma, chronic obstructive pulmonary disease, pneumothorax, hemoptysis, pneumonia, and pleural infections), cardiovascular disease (including acute coronary syndrome, congestive heart failure, cardiogenic shock, arrhythmia, and cardiac arrest), sepsis (including all septic conditions, but excluding pneumonia and pleural infections), gastrointestinal and hepatobiliary disease, neurologic disease (including seizures, intracranial hemorrhage, stroke, and coma), renal disease, endocrine disease, and others (not belonging to the above categories). We used in-hospital mortality as an outcome measurement and it was defined as death due to any cause during hospitalization. In addition, we collect the data of ventilator dependency which was defined if the patients cannot be successfully weaned off mechanical ventilator during this episode of hospitalization (including intensive care unit, respiratory care center, and ward). Data were collected on a routine basis, and the analysis was conducted retrospectively. The study was approved by the institutional review board of Chi Mei Medical Center, and informed consent was waived (10509-L02).

### Statistical analysis

Continuous variables including age, Glasgow Coma Scale score, APACHE II score, serum albumin, hemoglobin, BUN, and creatinine levels at admission, LOS, and duration of MV use are expressed as means ± standard deviations or median and interquartile range (IQR) depending on the nature and distribution of the variables. Categorical variables including gender, category of ICU, comorbidity, and organ dysfunction are expressed as count and percentage. Differences in baseline characteristics and clinical variables between the survival and mortality groups were evaluated using Student's *t*-test or Manny-Whitney U rank test for continuous variables and Pearson chi-square tests for categorical variables. A multivariable logistic regression model was constructed from baseline characteristics and clinical variables with p-values <0.05 as candidates. To determine the final prediction model, a stepwise model-selection procedure, in which all candidate variables were inserted until non-effects entered or an effect was removed from the backward elimination, was used to examine the association between predictive variables and the mortality rate and MV dependency using odds ratios (OR) with 95% confidence intervals (95% CI). All statistical analyses were conducted using the statistical package SPSS for Windows (Version 19.0, SPSS, Chicago, Il, USA), and a P value <.05 was considered to show statistical significance.

### Sponsor's role

None.
