# Correction: The outcomes and prognostic factors of acute respiratory failure in the patients 90 years old and over

**DOI:** 10.18632/oncotarget.24864

**Published:** 2018-03-20

**Authors:** Wan-Ling Chen, Chin-Ming Chen, Shu-Chen Kung, Ching-Min Wang, Chih-Cheng Lai, Chien-Ming Chao

**Affiliations:** ^1^ Department of Respiratory Therapy, Chi Mei Medical Center, Liouying, Tainan, Taiwan; ^2^ Department of Intensive Care Medicine, Chi Mei Medical Center, Liouying, Tainan, Taiwan; ^3^ Department of Recreation and Health-Care Management, Chia Nan University of Pharmacy & Science, Tainan, Taiwan; ^4^ Department of Internal Medicine, Chi Mei Medical Center, Liouying, Tainan, Taiwan; ^5^ Department of Intensive Care Medicine, Chi Mei Medical Center, Liouying, Tainan, Taiwan; ^6^ Department of Nursing, Min-Hwei College of Health Care Management, Tainan, Taiwan

**This article has been corrected:** The proper name of the institutions are as follows:

^**1**^ Department of Respiratory Therapy, Chi Mei Medical Center, Liouying, Tainan, Taiwan

^**2**^ Department of Intensive Care Medicine, Chi Mei Medical Center, Liouying, Tainan, Taiwan

^**3**^ Department of Recreation and Health-Care Management, Chia Nan University of Pharmacy & Science, Tainan, Taiwan

^**4**^ Department of Internal Medicine, Chi Mei Medical Center, Liouying, Tainan, Taiwan

^**5**^ Department of Intensive Care Medicine, Chi Mei Medical Center, Liouying, Tainan, Taiwan

^**6**^ Department of Nursing, Min-Hwei College of Health Care Management, Tainan, Taiwan

Original article: Oncotarget. 2018; 9:7197-7203. https://doi.org/10.18632/oncotarget.24051

